# Autochthonous canine leishmaniasis in Romania: neglected or (re)emerging?

**DOI:** 10.1186/1756-3305-7-135

**Published:** 2014-03-31

**Authors:** Viorica Mircean, Mirabela Oana Dumitrache, Mircea Mircean, Pompei Bolfa, Adriana Györke, Andrei Daniel Mihalca

**Affiliations:** 1 Department of Parasitology and Parasitic Diseases, University of Agricultural Sciences and Veterinary Medicine, Calea Mănăştur 3-5, Cluj-Napoca 400372, Cluj, Romania; 2 Department of Internal Medicine, University of Agricultural Sciences and Veterinary Medicine, Calea Mănăştur 3-5, Cluj-Napoca 400372, Cluj, Romania; 3 Pathology Department, University of Agricultural Sciences and Veterinary Medicine, Calea Mănăştur 3-5, Cluj-Napoca 400372, Cluj, Romania; 4 Department of Biomedical Sciences, Ross University School of Veterinary Medicine, PO Box 334, Basseterre, St. Kitts, West Indies

**Keywords:** *Leishmania infantum*, Dog, Romania, Canine leishmaniasis

## Abstract

Canine leishmaniasis is a vector-borne zoonotic disease caused by the protozoan parasite *Leishmania infantum*. In Romania between 1955 and 2013, no cases of human autochthonous visceral leishmaniasis were reported. Data regarding canine leishmaniasis is similarly scarce. Since the first report of clinical autochthonous canine leishmaniasis in 1935, there were only three sporadic reports of positive dogs all without any clinical signs. Our study reports the first clinical case of autochthonous canine leishmaniasis in the last 80 years, stressing the importance of a targeted surveillance of *Leishmania* infection, as infected dogs act as the primary reservoir for zoonotic visceral leishmaniasis.

## Findings

Human visceral leishmaniasis (VL) caused by *Leishmania infantum* is one of the most important emerging protozoan zoonosis. The parasite is transmitted by sand flies mainly from canine reservoirs to several vertebrates, including humans. Even though considered as a tropical disease, VL is endemic in nine European countries, with high prevalence of asymptomatic carriers in endemic foci [1].

The first case of autochthonous human leishmaniasis in Romania was reported in 1912 [2] and the first report of clinical autochthonous canine leishmaniasis was in 1935 [3]. Between 1944 and 1955, there were 26 autochthonous human case reports, all from southern Romania. Two of these were isolated cases (Prahova and Giurgiu Counties) [4,5] and one was an outbreak (Dolj County) [6]. Several studies were performed in the foci area in order to identify the reservoirs. Microscopical prevalence of *Leishmania* in dogs from Dolj was 1.2% [7]. In a neighboring County (Caraș-Severin) the prevalence in dogs was 2.2% [8]. However, all the positive dogs were asymptomatic.

No other cases of *Leishmania* infection were reported in Romania between 1969 and 1998. This was attributed to the mass use of insecticide between 1958 and 1964, during the malaria eradication programmes [4]. Since 1999, due to the increasing number of migrant workers to southern Mediterranean countries, 19 cases of imported VL were reported. In 2013, more than 80 years after the first case of autochthonous VL, another human case was reported in a patient from north-eastern Romania, with no history of travelling abroad but having travelled to the southern part of Romania [9]. Data regarding canine leishmaniasis is similarly scarce in Romania. In a recent study, four out of 138 dog serum samples were positive for *Leishmania* spp. infection by IFAT [10]. There are no recent data regarding species composition and geographical distribution of the sand fly fauna in Romania. A review of some older publications reports the presence of 8 species, among them *Phlebotomus perfiliewi* and *P. neglectus* being competent vectors for *L. infantum*[4].

**Figure 1:**
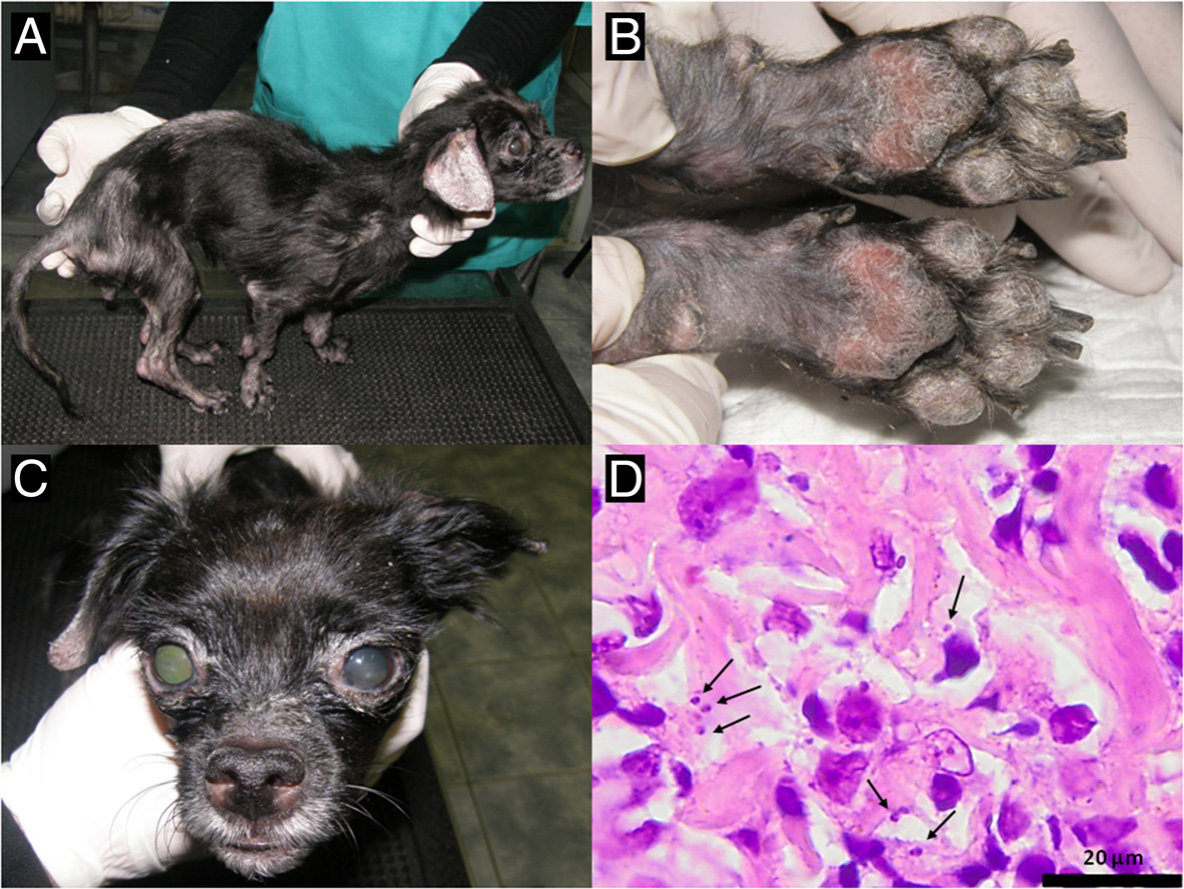
**Clinical and histopathological aspects in a 6 year old mixed breed dog with leishmaniasis. A**. Muscle waisting of the body muscles, exfoliative dermatitis, difuse alopecia; **B**. Footpads thickening and scaling, onychogryphosis; **C**. Nasal depigmentation, blepharitis, anterior uveitis; **D**. Skin biopsy: few round to oval, 2–3 micron protozoal amastigotes (arrows) located intrahistiocytic or extracellularly. Several small lymphocytes are also present in this section of the superficial dermis (hematoxylin and eosin stain).

Our letter reports the first clinical case of autochthonous canine leishmaniasis in the last 80 years. A 6 year old mixed-breed bitch from Vâlcea County (southern Romania), with no history of travelling and living outdoors was hospitalized at the Faculty of Veterinary Medicine Cluj-Napoca. The dog was brought in for the evaluation of a seborrhoeic, non-pruritic dermatitis, with a history of three months, initially this was found on the face and ear pinnae and then eventually covered all of the body, the dog also suffered from progressive weight loss and recurrent lameness. The clinical examination revealed generalized alopecia and psoriasiform scales (Figure 1A), thickening and scaling of the footpads and onychogryphosis (Figure 1B), bilateral uveitis and blepharitis (Figure 1C) and depigmentation of the nasal mucous membrane and conjunctiva. The complete blood count showed a mild non-regenerative anemia [RBC 5.61 × 10^12^/L, (reference interval 5.5 – 8.5 × 10^12^/L)], [hemoglobin 111 g/L (120 – 180 g/L)], [PCV 30% (37 – 55%)], polychromasia) and thrombocytopenia [51 ×10^9^/L (200–500 × 10^9^/L)]. An acute inflammatory condition was suggested by leukocytosis 19.45 ×10^9^/L [6–17 ×10^9^/L] and regenerative left shift. Serum biochemistry revealed hyperproteinemia 100.0 g/L [54.0 – 82.0 g/L], hyperglobulinemia 83.0 g/L [23.0 – 52.0 g/L] and hypoalbuminemia 18.0 g/L [25.0 – 44.0 g/L].

Skin biopsies from the ear pinnae, footpads and periorbital areas were collected for histological examination. Formalin-fixed paraffin-embedded tissues were sectioned and stained using hematoxylin-eosin (HE) and Giemsa methods. Microscopical examination revealed a nodular (periadnexial) to interstitial pattern of inflammation, primarily in the upper and mid-dermis, composed mainly of pale granular or foamy macrophages with less lymphocytes and plasma cells, concurrent with obliteration of sebaceous glands. Multifocal, around the blood vessels, there were accumulations of macrophages, lymphocytes, plasma cells and scant neutrophils. The epidermis was mildly hyperplastic and hyperkeratotic. Few amastigotes (2–4 μm) within the cytoplasm of histiocytes or extracellular (Figure 1D) were revealed. A rapid diagnostic test (FASTest®LEISH, sensitivity 98%; specificity 97%) for the qualitative detection of *L. infantum* IgG antibodies yielded a positive result.

The importance of our finding is related to the autochthonous nature of the clinical infection, which, despite not being the first, comes after a relatively long time frame since the previous one. Nevertheless, a targeted surveillance of *Leishmania* infection in dogs is not performed routinely in Romania. Moreover, the awareness of vets on canine leishmaniasis is relatively low, as many of them consider the disease absent (A.D. Mihalca pers. comm.). Due to this lack of clinical awareness, in the absence of extensive and long-term epidemiological surveys it is difficult to conclude if leishmaniasis is (re)emergent or simply neglected. However, as infected dogs act as the primary reservoir for zoonotic visceral leishmaniasis in Europe and the spread of canine leishmaniasis in continental regions of Europe represents a significant risk to human health, our report highlights the importance of permanent surveillance, mainly in the southern part of the country where all autochthonous cases have been reported so far.

## Competing interests

The authors declare that they have no competing interests.

## Authors' contributions

VM conducted the case and diagnosis approach. VM, MOD and AG performed clinical examination. MM carried out hematology and blood biochemistry. PB performed histological examination of the skin biopsy. AG prepared the figures. VM, MOD and ADM wrote the manuscript. All authors read and approved the final version of the manuscript.

## References

[B1] Gradoni L Epidemiological surveillance of leishmaniasis in the European Union: operational and research challenges Euro Surveill 2013 18 20539 23929176 10.2807/1560-7917.es2013.18.30.20539

[B2] Manicatide N Two cases of Kala-Azar observed in Romania [in french] Bull Sect Sci Acad Roum 1919–1920 105

[B3] Mihăilescu M Niciloff D Two cases of spontaneous canine leishmaniasis in Romania [in romanian] Arhiva vet 1934 26 43 53

[B4] Dancesco P Species of sandflies (Diptera: Psychodidae) in Romania, some aspects of their ecology and new capture stations [in french] Trav Mus Nat Hist Grigore Antipa 2008 51 185 199

[B5] Copăceanu P Motilică V Nicolaescu N Iliescu I The first case of visceral leishmaniasis in adults in RPR [in romanian] Rev Ig Microbiol Epid 1955 3 88 90 13350886

[B6] Minculescu M Bîrzu I Crețu S Iovănescu F Ionescu D Lupulescu V Michel G Pavlov S Rotaru A Rusovici I Zaharia C Reflections on the first outbreak of infantile leishmaniasis identified RPR [in romanian] Stud Cercet Inframicrobiol Parazitol III 1955 596 603 13324602

[B7] Lupașcu G Ciplea A Gherman I Contributions to the study of the reservoir of infection in the outbreak of leishmaniasis in Craiova region [in romanian] Stud Cercet Inframicrobiol Parzitol 1967 8 647 651

[B8] Lupașcu G Bossie A Ciplea A Costin P Research on the animal reservoir of Leishmania [in french] Arch Roum Pathol Exp 1968 28 29 35 5401159

[B9] Gogoaşe MG Teodorescu I Preda C Ionescu SC Two case reports on visceral leishmaniasis diagnosed in Romania Roum Arch Microbiol Immunol 2013 72 49 62 23947013

[B10] Hamel D Silaghi C Lescai D Pfister K Epidemiological aspects on vector-borne infections in stray and pet dogs from Romania and Hungary with focus on *Babesia* spp Parasitol Res 2012 110 1537 1545 10.1007/s00436-011-2659-y 21947342

